# Facial misfits accelerate stereotype-based associative learning

**DOI:** 10.1038/s41598-024-67770-8

**Published:** 2024-08-20

**Authors:** Marius Golubickis, Linn M. Persson, Johanna K. Falbén, Siew Hwee Seow, Parnian Jalalian, Yadvi Sharma, Margarita Ivanova, C. Neil Macrae

**Affiliations:** 1 School of Psychology, University of Aberdeen, King’s College, Aberdeen, AB24 3FX Scotland, UK; 2 Department of Psychology, University of Amsterdam, Amsterdam, The Netherlands

**Keywords:** Stereotyping, Learning, Prediction errors, Facial typicality, Person perception, Human behaviour, Psychology

## Abstract

Counterstereotypes challenge the deleterious effects that gender-typed beliefs exert on people’s occupational aspirations and lifestyle choices. Surprisingly, however, the critical issue of how readily unexpected person-related knowledge can be acquired remains poorly understood. Accordingly, in two experiments in which the facial appearance of targets was varied to manipulate goodness-of-stereotype-fit (i.e., high vs. low femininity/masculinity), here we used a probabilistic selection task to probe the rate at which counter-stereotypic and stereotypic individuals can be learned. Whether occupational (Expt. 1) or trait-related (Expt. 2) gender stereotypes were explored, a computational analysis yielded consistent results. Underscoring the potency of surprising information (i.e., facial misfits), knowledge acquisition was accelerated for unexpected compared to expected persons, both in counter-stereotypic and stereotypic learning contexts. These findings affirm predictive accounts of social perception and speak to the optimal characteristics of interventions designed to reduce stereotyping outside the laboratory.

## Introduction

Acquired through a combination of direct experience and media portrayals, gender stereotypes exert a pernicious influence on daily life^1^. From restricted educational and occupational ambitions to internalized convictions regarding acceptable modes of conduct, beliefs about the sexes shape thinking and doing in troublesome ways. For example, despite increased awareness of pervasive gender-based disparities, women continue to be underrepresented in positions of leadership and the science, technology, engineering, and mathematical (STEM) fields, while men are rarely encountered in communal roles in either healthcare or primary education^2,3^. Acknowledging the problems these inequities create, interest has naturally focused on devising interventions (e.g., commercials, job adverts, cartoons) with the potential to eliminate (or at least attenuate) gender stereotyping^4,5^. In this regard, one popular approach provides a practical pathway through which this objective can be achieved. Expose people to counterstereotypes—such as female construction workers and male nursery teachers—and constraining gender-related misconceptions may melt away.

Of course, for counterstereotypes to offset traditional gender-based beliefs a simple precondition needs to be satisfied, individuals must be receptive to the acquisition of expectancy-discrepant (i.e., counter-stereotypic) knowledge. Specifically, stereotype-violating persons should be easy to learn. But is this in fact the case? Several influential theoretical accounts of mental life give grounds for optimism (e.g., mismatch theory, predictive coding). The potency of unexpected information, as exemplified by counterstereotypes, is a cornerstone of psychological functioning^6–9^. Learning from previous experiences to generate predictions about future states of the world is one of the brain’s foremost capacities. Computationally, the precision of these predictions is enhanced when discrepancies are detected between anticipated and actual outcomes; that is, when prediction errors occur. Once generated, these prediction errors are used to update existing beliefs and craft new and improved forecasts. From this perspective, stereotypes can be characterized as predictive templates that furnish forecasts regarding the probable appearance, attributes, and proclivities of group members, with counterstereotypes grounded in sensory experiences that violate these expectations^9–11^.

As the acquisition of counter-stereotypic knowledge comprises the key ingredient of interventions designed to undermine gender-based beliefs, it is surprising that relatively little is known about the efficacy of this process. Recently, however, to inform understanding of this matter, work has explored how rapidly stereotype-related targets (i.e., stereotypes & counterstereotypes) can be learned^12^. Utilizing a modified probabilistic selection task (PST), participants were presented with three different pairs of female or male faces (AB, CD, EF) and, over the course of numerous choice selections (choose A or B), had to learn which person in each stimulus pair was most likely to prefer ballet or boxing as their favorite pastime^13,14^. Thus, the target selected on each trial comprised either a stereotype (i.e., female + ballet/male + boxing) or counterstereotype (i.e., female + boxing/male + ballet). Feedback was given after each selection to indicate whether the chosen face was correct or incorrect, but crucially this information was probabilistic. In AB trials, choosing A led to correct (i.e., positive) feedback in 80% of the trials, whereas B was accompanied by incorrect (i.e., negative) feedback in these trials (feedback was reversed for the remaining 20% of AB trials). Learning was more difficult for the other stimulus pairs, such that C was the correct response in 70% of CD trials, and E was the correct response in only 60% of EF trials. Thus, utilizing this methodology, stereotype-based associative learning was assessed under conditions of uncertainty.

Adopting a computational analysis to explore the dynamics of learning (i.e., Reinforcement Learning Drift Diffusion Model [RL-DDM] analysis), Falbén and colleagues’ findings were revealing. Underscoring the potency of prediction errors^6,7,9^, learning rates were faster for counterstereotypes compared to stereotypes. Specifically, learning was speeded when women preferred boxing (vs. ballet) and men favored ballet (vs. boxing). Significantly, however, although providing initial evidence that learning is facilitated for stereotype-incongruent individuals (i.e., prediction errors), Falbén et al. overlooked a fundamental facet of person perception with direct implications for knowledge acquisition—the degree to which a target’s facial appearance accords with prevailing stereotype-related preconceptions^15^. Not only is stereotyping driven by the classification of individuals into distinct social categories^16,17^, so too it is impacted by variability in the physical appearance of the members of these groups (i.e., within-group facial diversity). Individuals differ markedly in the extent to which their facial appearance matches stereotypic beliefs about the groups to which they belong, a factor that exerts considerable influence on person appraisal^15,18–20^. These effects, we suspect, may also extend to the efficiency of stereotype-based learning^12,21^.

For the most part, individuals with faces that best fit stereotype-based preconceptions (e.g., feminine women, masculine men) elicit elevated levels of stereotype activation and application^22–28^. For example, in the context of gender-typed occupations, the relative femininity/masculinity of faces impacts both candidate evaluations and hiring decisions, with outcomes determined by the degree to which role-related expectations and facial appearance correspond (i.e., stereotype-representative faces are preferred for gender-typed roles^29–31^. By extension, whether faces match or mismatch gender-based presumptions may also be an important determinant of stereotype-based learning. Specifically, whereas gender-atypical persons (e.g., low-feminine females) should be most surprising when encountered in stereotypic occupations (e.g., hairdressers), gender-typical (e.g., high-feminine females) individuals, in contrast, should be least expected when found in counter-stereotypic professions (e.g., plumbers). In other words, goodness-of-stereotype-fit determines the occupational settings in which people are most (or least) surprising (i.e., lack of fit model)^32^. If indeed the case, then by triggering prediction errors, facial misfits potentially have the capacity to enhance knowledge acquisition both in stereotypic and counter-stereotypic learning contexts, a possibility we explored in the current inquiry.

## Current research

Using a PST, here we investigated the effects that facial misfits exert on stereotype-based associative learning^12^. Participants were presented with three different pairs of female or male faces (AB, CD, EF), with each pairing comprising a high- and low-goodness-of-fit stereotype-related target (i.e., faces differed in femininity/masculinity). Over the course of numerous choice selections, and accompanying probabilistic feedback, their task was to learn which person in each stimulus pair was most likely to work as a beautician or builder (i.e., Expt. 1) or was affectionate or strong (i.e., Expt. 2). Thus, learning was probed in the context of both occupational and personality-related stereotypes^1^. To investigate the extent to which goodness-of-stereotype-fit influenced knowledge acquisition, an RL-DDM analysis was conducted on the data^33,34^.

Integrating sequential sampling and reinforcement learning models, the RL-DDM is useful as it identifies the processes that support decision-making and how these evolve as learning progresses^35^. This is achieved through the simultaneous hierarchical Bayesian modeling of response time and choice data. Crucially, a learning rate parameter (i.e., *η*) quantifies how quickly people learn, with larger values indicating enhanced updating from recently experienced outcomes; thus, faster learning. A scaling parameter (i.e., drift rate, *v*) measures sensitivity to feedback, taking both the expected outcome and speed of evidence accumulation into account. The model also establishes how much evidence is needed to generate a decision (i.e., boundary separation, *a*), with larger values indicating increased response caution. Across both experiments, in terms of the theoretically important learning rate parameter, we hypothesized that knowledge acquisition would be faster for facial misfits in both counter-stereotypic and stereotypic learning contexts. Specifically, whereas learning would be accelerated for faces displaying high-goodness-of-fit when counterstereotypes were to be learned, it would be speeded for faces exhibiting low-goodness-of-fit when stereotypes comprised the stimuli of interest.

## Experiment 1: beautician or builder?

### Methods and materials

#### Participants

One hundred and ten participants (75 females, 33 males, 2 other; *M*_age_ = 26.66, *SD* = 3.88), with normal or corrected-to-normal visual acuity, took part in the research. Seven participants (5 female, 2 male) were excluded due to failures in the attention checks^36^. Data collection was conducted online using Prolific Academic (www.prolific.co), with each participant receiving compensation at the rate of £8 (∼$10) per hour. Informed consent was obtained from participants prior to the commencement of the experiment and the protocol was reviewed and approved by the Ethics Committee at the School of Psychology, University of Aberdeen. The experiment was performed in accordance with the relevant guidelines and regulations and had a 2 (Learning Context: stereotypic or counter-stereotypic) X 2 (Goodness-of-Fit: high or low) mixed design with repeated measures on the second factor. To detect a significant effect (i.e., Learning Context X Goodness-of-Fit interaction) with a large effect size (i.e., *d* = 0.80)^12,21^, a sample of one hundred and three participants afforded 97% power (PANGEA, v 0.0.2).

#### Stimuli and procedure

**Figure 1:**
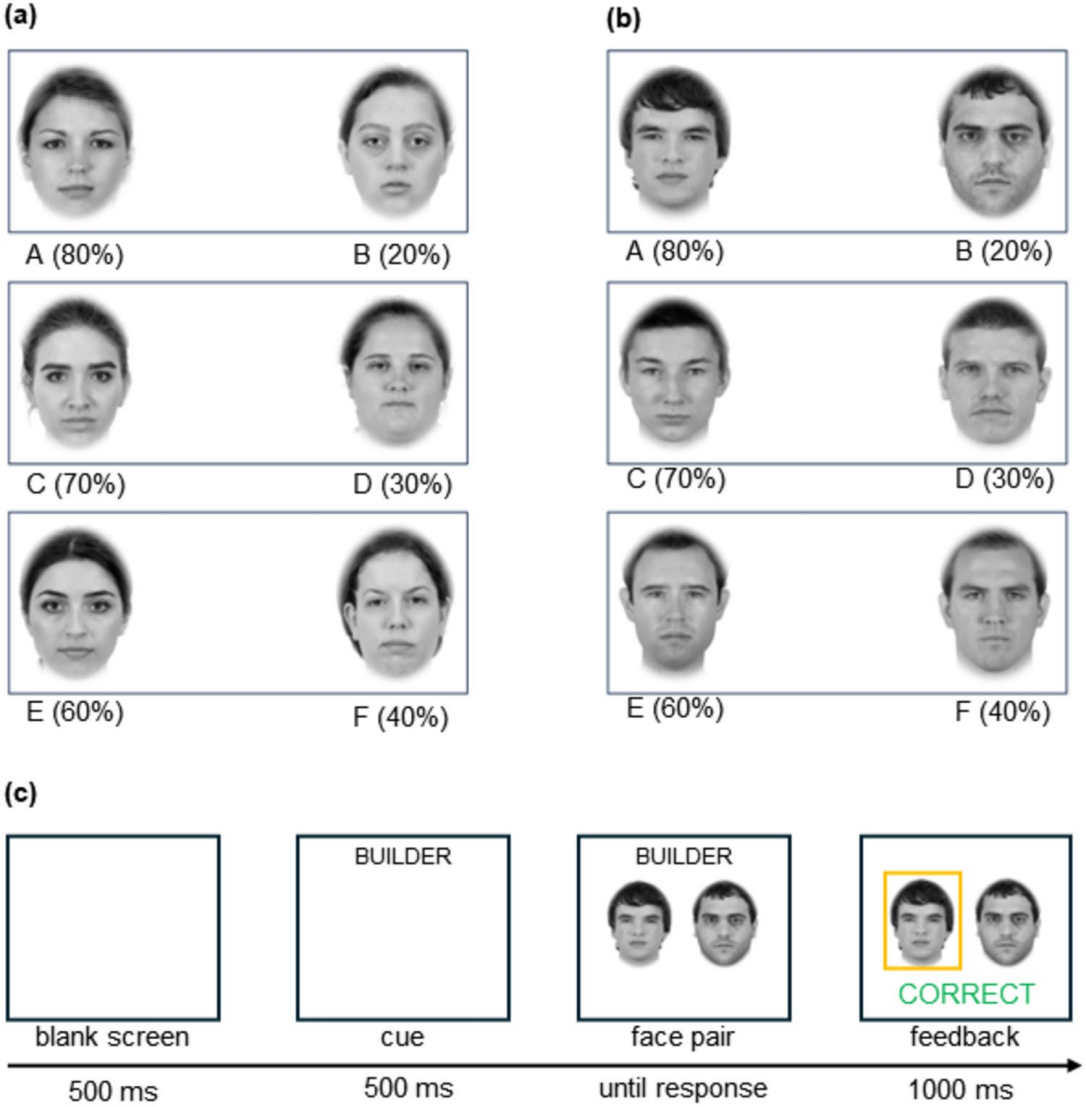
To-be-learned female (Panel **a**) and male (Panel **b**) faces and the probabilities of correct feedback during the probabilistic selection task. In Panel (**a**), ACE/BDF are high/low goodness-of-fit. In Panel (**b**), ACE/BDF are low/high goodness-of-fit. Example of an experimental trial (Panel **c**).

Participants completed a PST in which three pairs of same-sex faces (labeled AB, CD, and EF, see Fig. 1) were displayed, with each pairing comprising a high- and a low-goodness-of-fit stereotype-related face. Through the course of numerous choice selections, participants were required to learn, with the aid of feedback (i.e., correct or incorrect), which person in each pair was most likely to work as either a beautician or a builder. The occupation of the target was signaled by a cue (i.e., the labels “BEAUTICIAN” or “BUILDER”) which appeared on a trial-by-trial basis before each face pair was presented. Participants were randomly assigned to learning contexts that either confirmed (i.e., the beautician label always preceded female faces and the builder label always preceded male faces—learn stereotypes) or disconfirmed (i.e., the beautician label always preceded male faces and the builder label always preceded female faces—learn counterstereotypes) traditional gender stereotypes^1^. During the PST, half of the participants experienced positive reinforcement (i.e., correct feedback) when they selected high-goodness-of-fit female faces and low-goodness-of-fit male faces, whereas the others received positive reinforcement when low-goodness-of-fit female faces and high-goodness-of-fit male faces were chosen.

The probabilities indicating which face was most likely to be correct followed the standard version of the PST^13,14^. Specifically, for the AB pair, A was 80% likely to be correct (20% for B), for the CD pair, C was 70% likely to be correct (30% for D), and finally, for the EF pair, E was 60% likely to be correct (40% for F). Over many choice selections, participants learned which person in each pairing was most likely to be correct (i.e., A, C, E rather than B, D, F) based on the feedback provided. The task was completed when participants reached sufficient levels of accuracy for each pairing (i.e., AB, 60% or above; CD, 55% or above; EF, 50% or above)^13,14^. Each trial began with the presentation of a cue (i.e., BEAUTICIAN or BUILDER) for 500 ms, followed by a face pair that remained on the screen until the participant made a response (see Fig. 1). After the participant selected one of the faces, both verbal (i.e., the word ‘Correct’ in green or ‘Incorrect’ in red) and auditory (i.e., a high-pitched beep for a correct response or a low-pitched beep for an incorrect response) feedback were presented for 1000 ms, followed by a blank screen for 500 ms, after which the next trial commenced. Participants had to select a face by pressing the appropriate button on the keyboard (i.e., ‘A’ for the face on the left side of the screen, ‘L’ for the face on the right side of the screen). The faces in each pair were equally likely to be presented on the left or right side of the screen.

The faces (6 female & 6 male) were taken from the Chicago Face Database^37^, were 150 mm × 176 mm in size, grayscale, and depicted young adults aged 20–30 years. For females, 3 faces were high (7-point scale: *M* = 5.55, *SD* = 0.02) and 3 faces were low (*M* = 3.65, *SD* = 0.28) in femininity (*t*(4) = 11.86, *p* < 0.001, *d* = 9.69). For males, 3 faces were high (*M* = 5.17, *SD* = 0.15) and 3 faces were low (*M* = 3.62, *SD* = 0.16) in masculinity (*t*(4) = 12.06, *p* < 0.001, *d* = 9.85). The experiment was conducted using Inquisit Web. Participants completed blocks of 120 trials in which each of the face pairs appeared randomly, equally often, until accuracy reached a satisfactory level. The maximum number of learning blocks was set to four (i.e., 480 trials in total) if the participant did not reach satisfactory levels of accuracy earlier in the task^14^. On completion of the experiment, participants were debriefed and thanked.

#### Analytic approach

To test the hypothesis that learning would be speeded for unexpected targets (i.e., facial misfits), an RL-DDM analysis was conducted on the data^35,38^. The RL-DDM is a computational tool that merges the principles of reinforcement learning (RL) with the drift–diffusion model (DDM^39^). This hybrid approach yields a sophisticated account of how individuals accumulate evidence in support of decisions through the adaptation of their choice selections based on feedback provided during learning. Specifically, as participants navigate the PST, the RL component of the model operates in a value-based manner, such that each choice is assigned an expected value reflecting the cumulative feedback history. This value-based approach is crucial in guiding decision-making, with choices with higher (vs. lower) expected values being favored. In this respect, the model’s learning rate parameter (*η*) quantifies how quickly participants adapt their subjective expected values in the presence of new information. A higher learning rate indicates rapid adjustment to recent feedback (i.e., speeded learning), whereas a lower rate signals slower updating.

In the initial phases of the PST when stimuli are unfamiliar and thus lack associative strength, the model predicts slower responses due to a smaller difference in the expected values between choices. However, as feedback is received, expected values are updated and the disparity between the values associated with different stimuli grow (i.e., positively vs. negatively reinforced faces), leading to faster and more accurate decision-making. The degree to which the learned expected values of stimuli are integrated into the evidence accumulation process is captured by the model’s drift rate scaling (*v*_*scaling*_) parameter, which indicates the speed of information sampling and confident use of acquired information (i.e., sensitivity to expected rewards). In other words, *v*_scaling_ reflects the degree to which the expected values of choices influence the evidence accumulation process, such that higher values suggest the efficient utilization of knowledge during decision-making and are associated with the exploitation (vs. exploration) of choice selections^34^.

Like other sequential sampling models (SSMs), the RL-DDM posits that evidence accumulates in favor of each choice option (e.g., face A vs. face B) until a critical evidential threshold is reached, when a response is then made. This threshold, represented by the model’s boundary separation (*a*) parameter, embodies the speed-accuracy trade-offs inherent in decision-making. For example, a conservative decision-making style, characterized by higher evidential requirements, would typically result in slower, but more accurate, responses. Finally, the model’s non-decision time (*t*_0_) parameter accounts for the time consumed by processes other than decision-making, including stimulus encoding and response execution. Through the integrated analysis of these various parameters, the RL-DDM furnishes a comprehensive model of the decision-making process during learning^35,38^.

To identify the processes underpinning learning and decision-making, we compared three distinct models. The first model assumed that the learning context—whether stereotypic or counter-stereotypic—was the sole driver of cognitive variance. As such, this model allowed the learning rate (*η*), drift rate (*v*_*scaling*_), and boundary separation (*a*) parameters to vary only as a function of learning context (i.e., goodness-of-fit did not influence performance). This model is rooted in the presumption that biases tied to stereotype-congruency dictate the learning trajectory and choice behavior^12^. The second model shifted focus, hypothesizing that goodness-of-fit was the sole determinant of behavior, thus learning rate (*η*), drift rate (*v*_*scaling*_), and boundary separation (*a*) varied only as a function of this factor (i.e., the learning context did not influence performance). This model explored the possibility that stimulus-based differences alone could account for variations in learning and decision-making^26,40,41^. Finally, an integrative model that considered the joint influence of the learning context and goodness-of-fit on the learning rate (*η*), drift rate (*v*_*scaling*_), and boundary separation (*a*) was estimated. Theoretically, this model is grounded on the assumption that the cognitive processes underlying stereotype-based associative learning are multifaceted, reflecting the operation of both top-down and bottom-up factors^18,19^.

#### Results

Models were estimated by fitting the data (i.e., choices and latencies) to a reinforcement learning extension of the Bayesian hierarchical drift diffusion toolbox (HDDMrl)^34,42^. All models were response-coded, such that the upper threshold corresponded to responses to stimuli that were positively reinforced (i.e., faces corresponding to the letters A, C, & E) and the lower threshold to stimuli that were negatively reinforced (i.e., faces corresponding to the letters B, D, & F). Bayesian posterior distributions were modeled using a Markov chain Monte Carlo (MCMC) with 10,000 samples (including 5,000 burn). For model comparison, the Deviance Information Criterion (DIC) was used as this approach is routinely adopted when comparing hierarchical Bayesian models^43^. Lower DIC values favor models with the highest likelihood and least number of parameters. This revealed better fit for the integrative model (DIC: 82,107) compared to either the learning context (DIC: 82,290) or goodness-of-fit model (DIC: 82,197). This model also converged well across three chains of 5,000 samples and 500 burn (Ȓ = 1.003)^44^. To further evaluate the best fitting model, a Posterior Predictive Check (PPC) was performed^34,42^. Using the posterior distributions from the integrative model, 50 simulated data sets were estimated. The quality of model fit was then gauged by comparing the simulated and observed data for choice proportions and decision times for each stimulus pair. A visual review of the PPC revealed a good model fit (see Supplementary Material).

Examination of the posterior distributions for the best fitting model yielded differences in learning rate (*η*), drift rate scaling (*v*_scaling_), and boundary separation (*a*).

**Figure 2:**
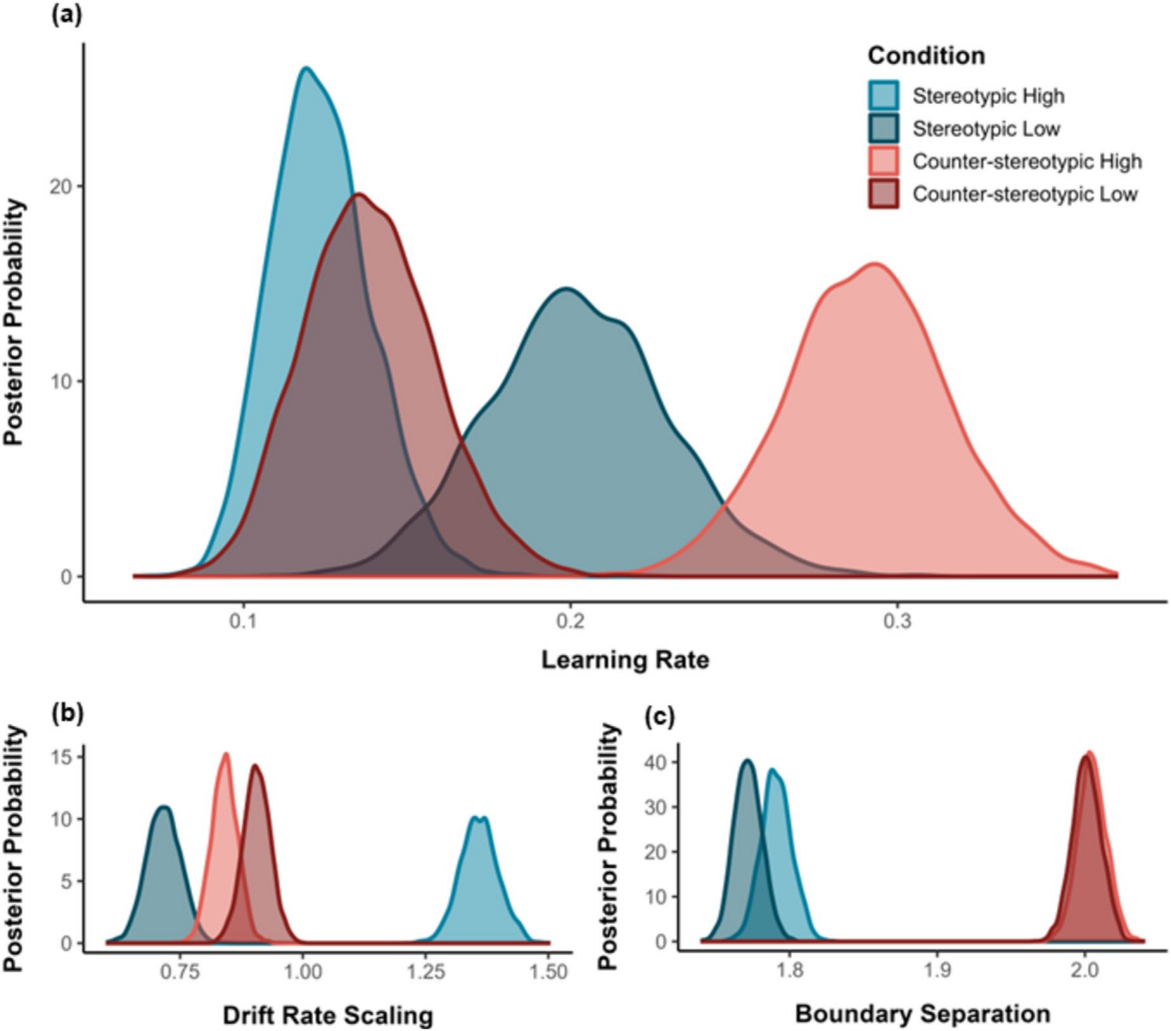
Mean posterior parameter distributions as a function of Learning Context and Goodness-of-Stereotype-Fit (Panel **a** = learning rate, Panel **b** = drift rate scaling, Panel **c** = boundary separation).

*Learning Rate (η)*. For high-goodness-of-fit faces, comparison of the posterior distributions furnished evidence that learning was faster for counterstereotypes compared to stereotypes (*p*_Bayes_[counterstereotypes > stereotypes] < 0.001, BF_10_ > 1000). This effect was reversed for low-goodness-of-fit faces, such that learning was speeded for stereotypes compared to counterstereotypes (*p*_Bayes_[counterstereotypes < stereotypes] = 0.031, BF_10_ = 31). Thus, knowledge acquisition was accelerated for unexpected individuals in both counter-stereotypic and stereotypic learning contexts (see Fig. 2, Panel a).

*Drift Rate Scaling (v*_*scaling*_*).* The analysis provided strong evidence that, for high-goodness-of-fit faces, drift rate scaling was larger for stereotypes compared to counterstereotypes (*p*_Bayes_[counterstereotypes < stereotypes] < 0.001, BF_10_ > 1000). For low-goodness-of-fit faces, in contrast, drift rate scaling was larger for counterstereotypes than stereotypes (*p*_Bayes_[counterstereotypes > stereotypes] < 0.001, BF_10_ > 1000). This indicates that reward sensitivity was greater for expected (vs. unexpected) persons in both learning contexts (see Fig. 2, Panel b).

*Boundary Separation (a).* The analysis yielded extremely strong evidence that, for high-goodness-of-fit faces, boundary separation was larger for counterstereotypes compared to stereotypes (*p*_Bayes_[counterstereotypes > stereotypes] < 0.001, BF_10_ > 1000). A similar effect was observed for low-goodness-of-fit faces (*p*_Bayes_[counterstereotypes > stereotypes] < 0.001, BF_10_ > 1000). Thus, regardless of facial appearance, response caution was larger in a counter-stereotypic (vs. stereotypic) learning context (see Fig. 2, Panel c).

**Figure 3:**
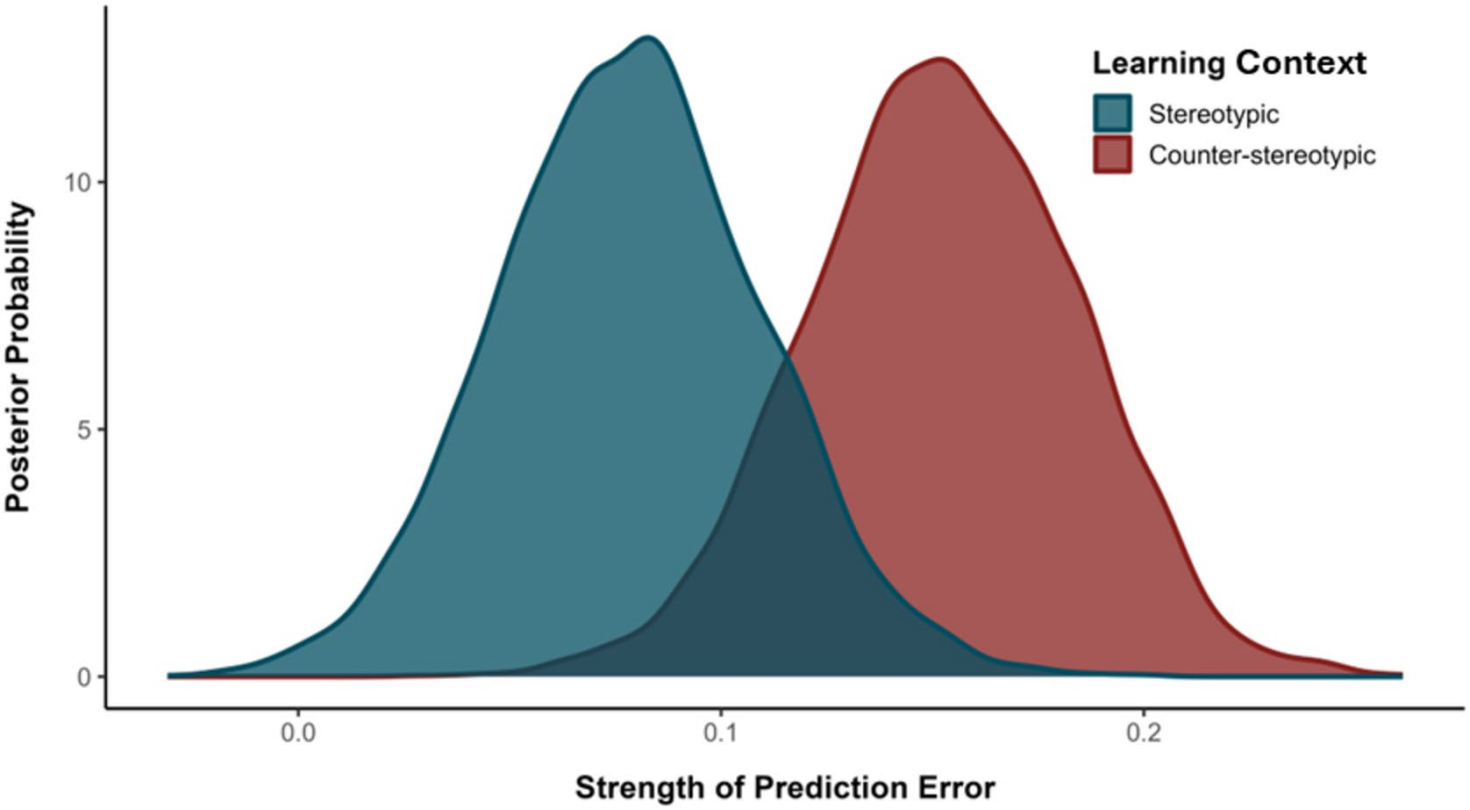
Strength of prediction error as a function of Learning Context.

*Comparing Prediction Errors Across Learning Contexts*. To probe the extent to which different types of prediction error (i.e., surprising counterstereotypes vs. surprising stereotypes) influence stereotype-based associative learning, we conducted an additional analysis focusing on the respective learning rates in each task context. Specifically, we subtracted the learning rates of high- from low-goodness-of-fit faces in stereotypical contexts (in which low-goodness-of-fit faces should be most surprising), and low- from high-goodness-of-fit faces in counter-stereotypical contexts (in which high-goodness-of-fit faces should be most surprising). In so doing, this yielded two distinct scores which measured the relative difference in surprise in each learning context. Comparison of these scores revealed the greater potency of facial misfits (i.e., prediction errors) in counter-stereotypical compared to stereotypical task contexts (*p*_*Bayes*_ = 0.11, BF_10_ = 8; see Fig. 3).

#### Discussion

The results of Experiment 1 confirmed that facial misfits accelerate stereotype-based associative learning. Whereas learning was speeded for high-goodness-of-fit faces when counterstereotypes were to be acquired, it was enhanced for low-goodness-of-fit faces when stereotypes comprised the to-be-learned material. That is, knowledge acquisition was accelerated for unexpected persons in both counter-stereotypic and stereotypic learning contexts, although the former effect was most pronounced. The objective of our next experiment was straightforward. To attempt to replicate these effects exploring a different aspect of gender stereotypes, personality characteristics associated with women and men^45^.

## Experiment 2: affectionate or strong?

### Methods and materials

#### Participants

As in Experiment 1, one hundred and ten participants (73 females, 36 males, 1 other; *M*_age_ = 25.39, *SD* = 3.14), with normal or corrected-to-normal visual acuity, took part in the research. Three participants (2 female, 1 male) were excluded due to failures in the attention checks^36^. Data collection was conducted online using Prolific Academic (www.prolific.co), with each participant receiving compensation at the rate of £8 (∼$10) per hour. Informed consent was obtained from participants prior to the commencement of the experiment and the protocol was reviewed and approved by the Ethics Committee at the School of Psychology, University of Aberdeen. The experiment was performed in accordance with the relevant guidelines and regulations and had a 2 (Learning Context: stereotypic or counter-stereotypic) X 2 (Goodness-of-Fit: high or low) mixed design with repeated measures on the second factor. The sample size calculation was as in Experiment 1.

#### Stimuli and procedure

The task closely followed Experiment 1, but with a single modification. On this occasion, participants were required to learn, on a trial-by-trial basis, which person in each pair was most likely to be affectionate (i.e., female stereotypic, male counter-stereotypic) or strong (i.e., female counter-stereotypic, male stereotypic). In all other respects, the procedure was identical to Experiment 1.

#### Results

Adopting the analytical approach applied in Experiment 1, data were submitted to a RL-DDM analysis. As previously, fit was better for the integrative (DIC: 84,126) compared to either the learning context (DIC: 84,336) or goodness-of-fit (DIC: 84,279) model. This model also converged well across three chains of 5,000 samples and 500 burn (Ȓ = 1.004)^44^. An additional PPC revealed a good model fit (see Supplementary Material). Examination of the posterior distributions for the best fitting model yielded differences in learning rate (*η*), drift rate scaling (*v*_scaling_), and boundary separation (*a*).

**Figure 4:**
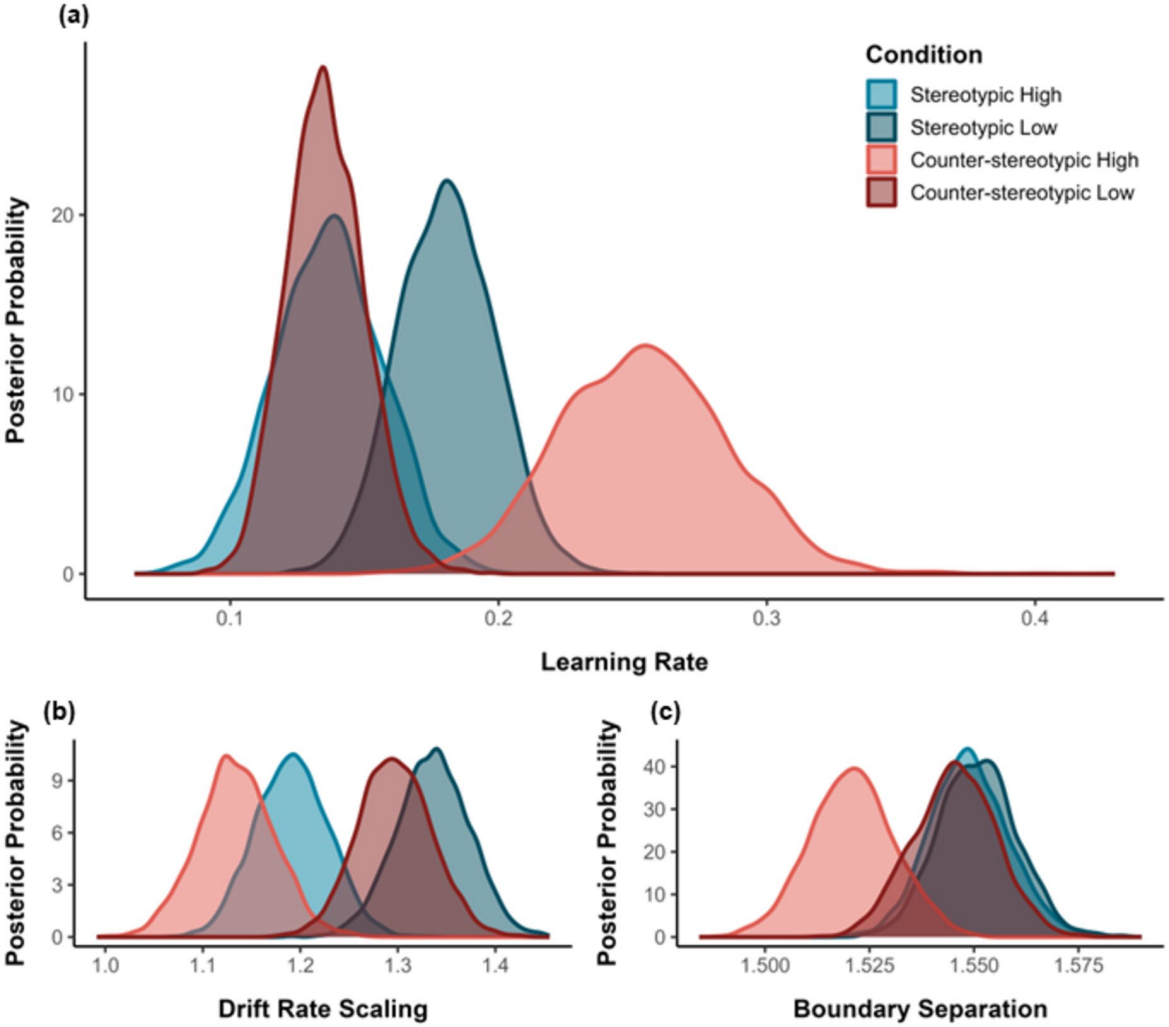
Mean posterior parameter distributions as a function of Learning Context and Goodness-of-Stereotype-Fit (Panel **a** = learning rate, Panel **b** = drift rate scaling, Panel **c** = boundary separation).

*Learning Rate (η)*. For high-goodness-of-fit faces, comparison of the posterior distributions provided evidence that learning was faster for counterstereotypes compared to stereotypes (*p*_Bayes_[counterstereotypes > stereotypes] = 0.002, BF_10_ = 499). This effect was reversed for low-goodness-of-fit faces, such that learning was accelerated for stereotypes compared to counterstereotypes (*p*_Bayes_[counterstereotypes < stereotypes] = 0.016, BF_10_ = 62). Thus, knowledge acquisition was speeded for unexpected persons in both learning contexts (see Fig. 4, Panel a).

*Drift Rate Scaling (v*_*scaling*_*).* The analysis provided evidence that, for high-goodness-of-fit faces, drift rate scaling was larger for stereotypes compared to counterstereotypes (*p*_Bayes_[stereotypes > counterstereotypes] < 0.193, BF_10_ = 4). A similar effect was observed for low-goodness-of-fit faces (*p*_Bayes_[stereotypes > counterstereotypes] < 0.220, BF_10_ = 4). This indicates that reward sensitivity was greater in a stereotypic (vs. counter-stereotypic) learning context (see Fig. 4, Panel b).

*Boundary Separation (a).* The analysis yielded extremely strong evidence that, for high-goodness-of-fit faces, boundary separation was larger for stereotypes compared to counterstereotypes (*p*_Bayes_[counterstereotypes < stereotypes] = 0.004, BF_10_ = 249). A similar but weaker effect was observed for low-goodness-of-fit faces (*p*_Bayes_[counterstereotypes < stereotypes] = 0.253, BF_10_ = 3). Thus, regardless of facial appearance, response caution was greater in a stereotypic (vs. counter-stereotypic) learning context (see Fig. 4, Panel c).

**Figure 5:**
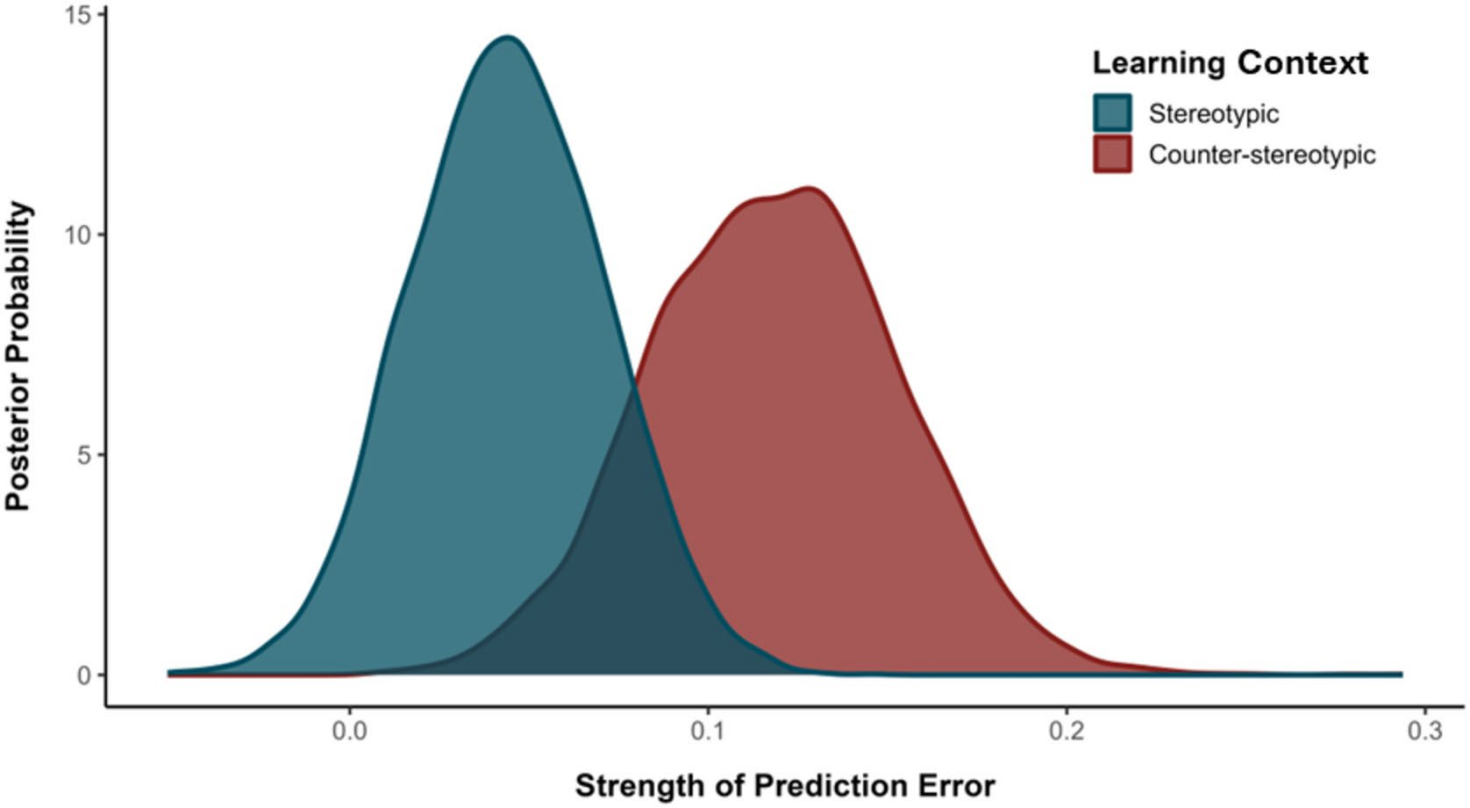
Strength of prediction error as a function of Learning Context.

*Comparing Prediction Errors Across Learning Contexts*. Comparison of the prediction errors in each task setting revealed the greater influence of facial misfits in counter-stereotypical compared to stereotypical contexts (*p*_*Bayes*_ = 0.12, BF_10_ = 7; see Fig. 5).

#### Discussion

In terms of the critical learning rates, the results of Experiment 2 directly replicated those observed previously. Knowledge acquisition was accelerated for facial misfits (i.e., prediction errors) in both counter-stereotypic and stereotypic learning contexts. As in Experiment 1, prediction errors were most potent in counter-stereotypic (vs. stereotypic) task settings.

## General discussion

When learning the identity of stereotype-related persons, facial appearance matters^15,18,19^. Across two experiments exploring different facets of gender stereotypes (i.e., occupational & trait-based beliefs), knowledge acquisition was faster for unexpected compared to expected targets in both counter-stereotypic and stereotypic learning contexts^12^. Specifically, learning was accelerated for high-goodness-of-fit faces when counterstereotypes were to be acquired, but for low-goodness-of-fit faces when stereotypes comprised the to-be-learned material. These findings confirm that facial appearance moderates the efficiency of stereotype-based associative learning, a finding with important implications for interventions designed to attenuate the deleterious effects of gender-typed beliefs outside the laboratory^5,46^. Notably, if associative learning enhances the acquisition of surprising counterstereotypes and stereotypes, this provides an important pathway through which gender-typed beliefs can be updated following the generation of erroneous stereotype-based predictions^10^.

To date, strategies conceived to overcome gender stereotyping have focused largely on presenting counter-stereotypic between-group role models to the audience of interest, usually (although not always) young children or adolescents^4,5^. For example, among girls aged 4–10 years, exposure to role-incongruent exemplars has been shown to reduce both their gender stereotypes and traditional attitudes toward women^47,48^. By highlighting that associative learning is sensitive to the facial appearance of counterstereotypes, the current findings offer valuable insights into the approaches best suited to reduce stereotyping^49^. To maximize the impact (e.g., learning) of between-group counterstereotypes in stereotypic landscapes, role-incongruent individuals should ideally possess gender-typical facial features. For instance, to undermine the belief that only men can be employed as motor mechanics, highly feminine (vs. less feminine) women should be depicted fixing vehicles in auto-repair workshops^4,5^. That is, counterstereotypes should violate pre-existing stereotypes based on both their group membership and facial appearance. Together with the demonstration that goodness-of-stereotype fit affects evaluations of women and men^29,30^, the current findings underscore the significance of facial appearance to the acquisition of person-related knowledge^15,18,19^.

Importantly, here we also considered how another class of facial misfits potentially influence associative learning. Specifically, individuals confirming the relevant stereotype based on their sex, but who display unexpected facial characteristics (i.e., surprising stereotypes). As predicted, these ill-fitting persons were learned more rapidly than their facially congruent counterparts, thereby suggesting another possible pathway through which gender stereotyping can be attenuated. Rather than defining role-incongruence based on sex categorization alone, attention should additionally be directed to gender-congruent targets (i.e., stereotypes) who are surprising by dint of their facial appearance^31^. Although such individuals clearly do not overcome traditional gender-typed beliefs, they nevertheless signal that a broad spectrum of women and men can be employed in stereotyped professions or exhibit gender-consistent attributes, thereby undermining the assumption that stereotypes must all look a certain way^46^. Although learning rates were fastest when individuals with gender-typical faces were counterstereotypes, here we showed that knowledge acquisition was also speeded (albeit to a lesser extent) when persons with low gender-typicality faces were stereotypes. In other words, unexpected persons accelerated knowledge acquisition in counter-stereotypic and stereotypic learning contexts. When designing future interventions, the potential applicability of these latter findings merits consideration.

Supported by a mind that is tuned to the acquisition of expectancy-discrepant knowledge, the current results resonate with predictive accounts of social perception^9–11,50^ and interactive theoretical models that underscore how the interplay between top-down (e.g., stereotype-based beliefs) and bottom-up (e.g., facial appearance) factors underpin person construal^18,19^. Whilst encountering stereotypes serves only to corroborate extant beliefs about the world, counterstereotypes contest prevailing wisdom and suggest that modified person-related forecasts may be required. Extending an established literature on the topic, not only are counterstereotypes attention-grabbing and memorable^16–18^, so too they enhance associative learning under conditions of uncertainty^12^. Of functional significance, person-related learning is accelerated when there is most to be learned. Of course, here we considered only gender stereotypes and disregarded important individual differences that impact person perception. As belief updating is modulated by the strength of prediction errors^6^, counterstereotypes should correspondingly exert greatest influence among individuals who endorse stereotype-related beliefs most strongly. That is, the effects of exposure to counterstereotypes should be most potent when incongruent individuals are least expected. In combination with the strength in which stereotypic convictions are endorsed, a useful line of future inquiry will be to explore associative learning across a diverse range of counterstereotypes (e.g., age, ethnicity, attractiveness), populations (e.g., children, adolescents, older adults), and task contexts (e.g., attentionally demanding settings). Work of this kind will be needed to establish the generality of the current effects.

Although the effects observed on learning rates were consistent across both Experiments 1 and 2, it should be noted that this was not the case for either the drift-rate scaling or boundary separation parameters. When occupational stereotypes were explored, reward sensitivity was greater for expected (vs. surprising) targets in both learning contexts and response caution was larger for counter-stereotypic (vs. stereotypic) decisions (Expt. 1). In contrast, when personality stereotypes comprised the gender-typed dimension of interest, reward sensitivity was greater in a stereotypic (vs. counter-stereotypic) learning context and response caution was larger for stereotypic (vs. counter-stereotypic) decisions (Expt. 2). These divergences speak to subtleties in the way in which different stereotype-related contents, in combination with facial appearance, potentially influence the cognitive operations that underpin decisional processing^12^. Unlike learning rates, sensitivity to anticipated rewards and the evidential requirements of response selection displayed greater variability as a function of the specific stereotype under investigation and the task context in which person-related knowledge was to be acquired. Thus, although unexpected persons (i.e., counterstereotypes) facilitate associative learning, under certain task conditions and stimulus configurations, stereotypes also differentially modulate components of decisional processing.

A limitation of the current research was that counter-stereotypes were deemed to be equally potent for both female and male targets (i.e., strong woman = affectionate man). This, however, is likely to be an oversimplification. Indeed, as a function of societal changes, personal experiences, and prior beliefs, there may be considerable variability in the extent to which people find counter-stereotypes to be surprising^1,5^. For example, men displaying affection may seem considerably less likely than women showing strength. Crucially, such an observation does not undermine the current findings, but rather highlights the intricacies and complexities of stereotype-based associative learning and the need for additional research employing a diverse range of stereotype-related contents. It is possible that the anatomy of decision-making may be quite distinct for different expressions of gender stereotypes^16–19^. These nuances aside, however, the consistency of the learning rate effects across both experiments indicate that the current results are robust and not an artifact of model parameter interdependencies.

Notwithstanding tactics that can be employed to preserve stereotypes in the face of disconfirmation (e.g., subtyping)^51,52^, counterstereotypes are widely believed to serve as an antidote to egregious stereotype-related beliefs. Through targeted interventions, role-incongruent individuals have the capacity to eliminate (or dilute) the effects of restrictive stereotype-based life choices, resulting in an equitable distribution of the sexes in all corners of society^4,5^. For this to happen, however, people must be receptive to the acquisition of stereotype-discrepant knowledge and then use this information to update their beliefs about women and men^9–11,50^. Here we provided evidence for at least the first of these requirements. Underscoring the mind’s receptivity to surprising information, learning was accelerated for unexpected compared to expected individuals, an effect that emerged in counter-stereotypic and stereotypic task contexts. These findings affirm predictive accounts of person perception and speak to the optimal characteristics of interventions devised to challenge traditional stereotype-based beliefs.

## Data Availability

Data are available at https://osf.io/enmzv/.
